# Automatic Visual Acuity Estimation by Means of Computational Vascularity Biomarkers Using Oct Angiographies

**DOI:** 10.3390/s19214732

**Published:** 2019-10-31

**Authors:** Macarena Díaz, Marta Díez-Sotelo, Francisco Gómez-Ulla, Jorge Novo, Manuel Francisco G. Penedo, Marcos Ortega

**Affiliations:** 1 Grupo VARPA, Instituto de Investigación Biomédica de A Coruña (INIBIC), Universidade da Coruña, 15006 A Coruña, Spain; jnovo@udc.es (J.N.); mgpenedo@udc.es (M.F.G.P.); mortega@udc.es (M.O.); 2 Centro de Investigación CITIC, Universidade da Coruña, 15071 A Coruña, Spain; 3 Complejo Hospitalario Universitario de Santiago, 15706 Santiago de Compostela, Spain; martads_2@hotmail.com (M.D.-S.); franciscogomez-ulla@institutogomez-ulla.es (F.G.-U.); 4 Instituto Oftalmológico Gómez-Ulla, 15706 Santiago de Compostela, Spain

**Keywords:** Optical Coherence Tomography by Angiography, Visual Acuity, Retinal Vein Occlusion, Artificial Vision, biomarkers

## Abstract

Optical Coherence Tomography Angiography (OCTA) constitutes a new non-invasive ophthalmic image modality that allows the precise visualization of the micro-retinal vascularity that is commonly used to analyze the foveal region. Given that there are many systemic and eye diseases that affect the eye fundus and its vascularity, the analysis of that region is crucial to diagnose and estimate the vision loss. The Visual Acuity (VA) is typically measured manually, implying an exhaustive and time-consuming procedure. In this work, we propose a method that exploits the information of the OCTA images to automatically estimate the VA with an accurate error of 0.1713.

## 1. Introduction

In many clinical specialties, there exists a high variety of image modalities that clinicians typically use to analyze the patient’s condition and aid in the diagnostic process. Given the importance of technology at present, namely, its inclusion in different areas to improve an expert’s work, many computational systems have been implemented to facilitate this clinical processes [1,2,3]. This automatization of different clinical manual tasks provide solutions and improvements that are characterized by desirable properties that the expert cannot offer such as determinism, repeatability or objectivity. Regarding image analysis, over the years, many computational proposals have been presented based on computer vision techniques that are exploited to analyze relevant areas of the images of interest, measure relevant parameters and monitor their evolution and potential variations, which is the typical procedure that is used in many Computer Aided Diagnostic (CAD) systems.

In ophthalmology, different image modalities have increased in popularity over the last years. The color fundus retinography is the most traditional and widely extended ophthalmic image modality, that allows the direct visualization of the eye fundus and their possible pathological visible structures. Being an invasive capture technique, the Fluorescein Angiography (FA) provides an enhanced visualization of the retinal vascular structure by injecting a dye contrast into the patient. With the Optical Coherence Tomography (OCT), we can visualize and analyze volumetric scans of the inner retinal layers, non-invasively and in real time. Based on the OCT and the FA characteristics, a new image modality appeared, combining both the OCT benefits and the FA visualization: the Optical Coherence Tomography by Angiography (OCTA). OCTA [4] imaging constitutes the new ophthalmic image modality, and it is the first that allows an exhaustive and non-invasive vascular analysis of the retina. OCTA accurately represents the vascularity of the foveal region within the retinal layers. This representation is extracted based on the blood movement in the eye microvasculature. Given that numerous systematic and eye diseases are related with the microvascular circulation, OCTA constitutes a relevant new ophthalmic image modality that better aids the diagnosis and monitoring processes of these pathologies. As reference, diseases such as diabetes can generate a Diabetic Retinopathy (DR), which provokes microvascular problems in advanced stages of the disease, eventually producing total blindness in the worst case scenario. Also, the Retinal Vein Occlusion (RVO) produces a problematic loss of vascularity and a progressive vision loss.

With these representative examples among many others, it is clear that the retinal vascular integrity is related with the Visual Acuity (VA) [5,6,7,8] of the patients. Commonly, to estimate the VA of a given patient, it is necessary to perform different rudimentary tests [9]. In particular, these tests are normally related with the analysis of the ability of the patient to observe characteristic objects of different sizes and at different distances. Consequently, the final VA estimation is subjective whereas its process is tedious and slow. In that context, any technological contribution that avoids or facilitates this revision process is extremely desired in the ophthalmic field.

Given the potential of the OCTA image modality, over recent years, several clinical studies were published demonstrating its utility in the analysis of different vascular diseases. Hence, Balaratnasingam et al. [5] demonstrated in their study that the VA is correlated with the Foveal Avascular Zone (FAZ) in patients with DR and RVO diseases. Other clinical studies [6,7,8] related different manual biomarkers extracted from the OCTA images with the VA of the patient. Wei et al. [10] demonstrated the adequate repeatability of the vascular density as representative biomarker in healthy subjects in both macular and optic disk regions. Posteriorly, Vivien at al. [11], demonstrated the influence of the glaucoma disease over the vessel tree characteristics, obtaining as conclusions the absence of retinal vascularity in the pathological cases over a control group. Given its novelty, there exists a limited number of computational proposals to aid the diagnosis and monitoring of pathologies with this image modality. In fact, the existing works are mainly based on the extraction of characteristic biomarkers in the OCTA images or improving the image quality to facilitate its posterior analysis. As reference, Díaz et al. [12] briefly introduced a method to automatically extract and measure generally the Vascular Density (VD). Also, the authors proposed a method to extract and measure the FAZ [13] in healthy and DR OCTA images and they performed a FAZ circularity study in healthy and pathological images. Alam et al. [14] proposed different features extracted from the OCTA images, namely, the vessel tortuosity, the mean diameter of the blood vessels, the semi-automatic FAZ area, the FAZ irregularity and the density of the parafoveal avascular region using fractal dimensions. Guo et al. [15] segmented and quantified avascular ischemic zones with map probabilities that were extracted by a deep convolutional neural network. Wang et al. [16] proposed a method to produce mosaics using different overlapping OCTA images, increasing the wide field of vision for the posterior clinical analysis. Wei et al. [17] improved the quality of the OCTA scans in their work whereas Camino et al. [18] identified existing shadow artifacts that typically appear in the OCTA images to improve the visualization and facilitate their inspection.

Given that there are several clinical studies that demonstrate the correlation of the VA with different parameters of the OCTA images, and the complexity of the VA manual calculation, a faster and more reliable alternative to automatically determinate this measurement is desired. Nowadays, no automatic approach is available that obtains the VA of the patient without the clinical intervention. For this reason, we propose a novel method to automatically estimate the VA of a patient using biomarkers that are directly extracted from the OCTA images. In fact, those biomarkers are barely used in existing state of the art works. The proposed method is mainly based on the extraction of two representative groups of biomarkers over the OCTA images and their posterior adaptation to be used as input in a Support Vector Machine (SVM) to estimate the target VA. The first biomarker is the FAZ area [13], given the popularity of the FAZ characteristics in relation with the vision problems. The second group of biomarkers measure the VD. The VD represents an important metric of the OCTA images, given the quality and precision of the vascular visualization in this image modality. Also, its manual measurement is not feasible, given the necessity of labeling each pixel that could be considered as a vessel. In that line, the difficulty of obtaining a manual labeling imply the problem of performing the validation process by the comparison with an expert ground truth. As an alternative, we propose a method to extract the VD, comparing the results with the performance of a semi-automatic module that provides a capture device of reference. Finally, using these validated biomarkers, we perform the estimation of the VA using an adapted and tested SVM, demonstrating that both the automatic FAZ area and the VD extraction are reliable and that the VA can be predicted from the OCTA images.

In line with this, a preliminary version of this methodology has been included in a clinical study (Díez-Sotelo et al. [19]) about how the visual acuity is affected in a particular cohort of ophthalmological patients. Once the automatic proposal has demonstrated its potential in a real case, the main objective of this work is to perform an exhaustive analysis of the performance of the methodology analyzing its main components as well as the most relevant computational characteristics in order to have an optimized and generalized methodology.

The paper is organized as follows: Section 2 details the used dataset for validation purposes as well as the characteristics of the proposed methodology; Section 3 exposes the performed experiments and their motivations; and, finally, Section 4 discusses the obtained results and the contribution of our proposal to the state of the art.

## 2. Materials and Methods

**Figure 1:**
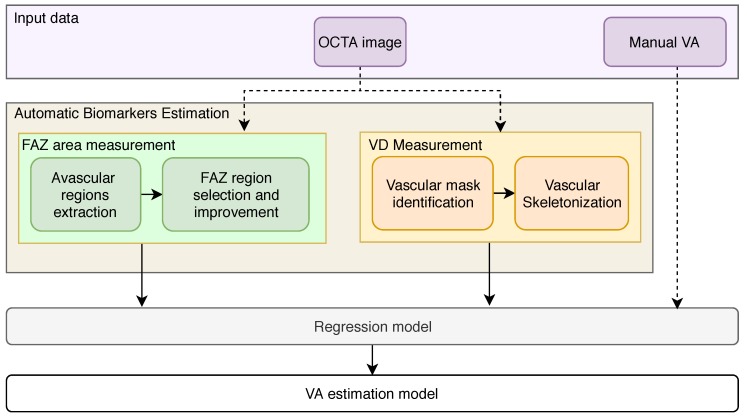
Main steps of the proposed methodology for the Visual Acuity (VA) estimation.

The proposed methodology is generally structured as presented in Figure 1, and is explained in the following sections. First of all, using the OCTA images as input, the method extract representative avascular and vascular biomarkers: the FAZ area is extracted identifying avascular regions followed by the FAZ region selection and the improvement of its contour [13]. Moreover, the VD is extracted using the skeletonization of a previously extracted vascular mask using an adapted thresholding process. Next, we adapt and train an SVM regression model to finally estimate the target VA using the extracted biomarkers as input features.

### 2.1. OCTA Image Acquisition

**Figure 2:**
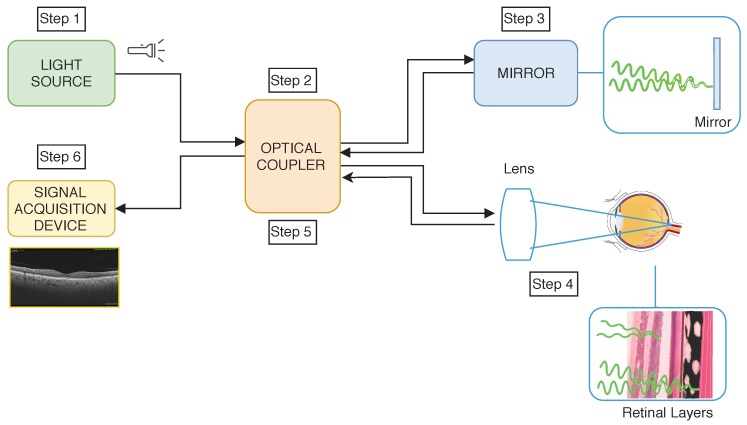
Graphical scheme of the Optical Coherence Tomography (OCT) image acquisition process.

The OCTA image acquisition process is based on the OCT extraction and the detection of the blood movement in successive OCT scans at the same depth of the eye fundus. In particular, it is generally based on the OCT acquisition process, which is performed following the process that we can graphically see in Figure 2 and that is explained below. In particular, OCT imaging is based on the interference of light over the retinal layers. For this reason, the first step implies the direct interference of light (Step 1 in Figure 2). The light is sent to the *optical coupler* and split into different optical fibers (Step 2 in Figure 2) that transmit the light to a mirror and to the retina, at the same time. The light that is reflected in the mirror returns in a constant way, and for this reason it is used as reference (Step 3 in Figure 2). By contrast, the reflected light in the retina differs in each retinal layer, as we can see in Step 4 of Figure 2. Then, the reflected lights from both the mirror and the retinal layers are compared. The difference between the light strength of each layer and the reference is measured (Step 5 in Figure 2) to obtain the final signal that is represented as the OCT image before the processed step of the measured information.

**Figure 3:**
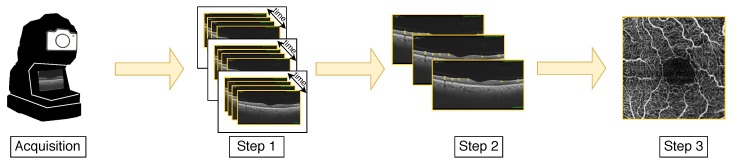
Graphical scheme of the Optical Coherence Tomography Angiography (OCTA) image acquisition process.

Once we capture the OCT images, the required steps to obtain the final target OCTA image are graphically represented in Figure 3. To do this, first, it is necessary to perform the explained OCT acquisition at the same positions and different progressive times (in the range of seconds). To obtain the spatial resolution of the OCTA images, it is also necessary the OCT acquisition at different positions from all the foveal zone. In Step 1 of Figure 3, we can see the graphical representation of the required data to obtain the OCTA images and their distribution: different groups of OCT image sets in the same place and in the nearby areas, using the 3-dimensional characteristics of the OCT images. For each group of OCT images that are extracted from the same position, the average is measured to obtain images with high quality and the blood flow measurement is made over the obtained OCT image (Step 2 in Figure 3) based on the differences over the OCT images at the same position. By using the blood flow in the nearest OCT scans, it is possible to construct the final target OCTA image (Step 3 in Figure 3).

### 2.2. Used Image Dataset

The methodology was validated using an image dataset that contains 860 OCTA images that were extracted by the capture device DRI OCT Triton; Topcon Corp. These OCTA images were obtained in progressive consultations of different patients that suffered RVO, with 3 months between each consultation. The dataset also includes the manual measurement of the VA of the patient in the pathological eye, extracted with the previously mentioned VA test procedure [9]. In particular, we organized the dataset into 215 samples, each one containing the information extracted from one patient consultation, including:

- The 4 different OCTA images that we use as reference: 3 mm ×3 mm Superficial Capillary Plexus (SCP), 3 mm ×3 mm Deep Capillary Plexus (DCP), 6 mm ×6 mm SCP and 6 mm ×6 mm DCP.
- The VA of the patient at the consultation, manually measured by a clinical specialist.

In summary, as indicated, the image dataset contains 860 images that we distributed into 215 samples, where each one represents the information of a patient in one ophthalmic consultation.

Moreover, in order to test the suitability and robustness of the automatically extracted vascular and avascular biomarkers, we further obtained the manual measurement of the FAZ area as well as the semi-automatic VD extraction that includes the used capture device of reference. Again, both tasks were performed by an expert clinician.

The used dataset was retrieved in clinical practice, where several images present some motion artifacts that are typical in this image modality. Despite that, these artifacts do not represent a significant proportion of the images to compromise the robustness of the method. In that line, OCTA images with severe deteriorations in the capturing process that are largely deformed were excluded from the study, as they represent images that are normally omitted from analysis by the expert clinicians given they constitute an unfeasible scenario to perform the manual measurement of the FAZ.

### 2.3. Automatic Estimation of the Avascular and Vascular Biomarkers

#### 2.3.1. FAZ Area Measurement

**Figure 4:**
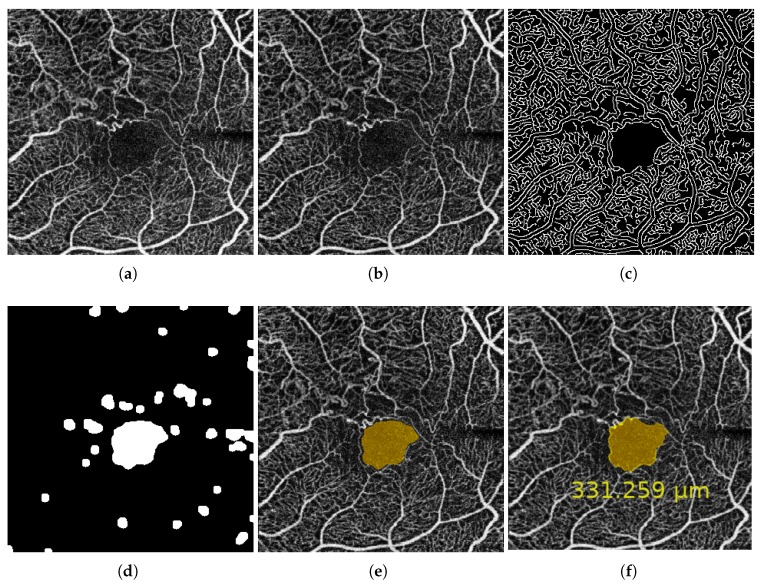
A representative example of the steps involved in obtaining the Foveal Avascular Zone (FAZ) segmentation. (**a**) Original image. (**b**) Enhanced image after applying a top-hat morphological operator. (**c**) Extracted edges from the enhanced image after applying an edge detector. (**d**) Obtained image after the application of morphological operators as opening and closing. (**e**) Result after removing small structures in (**d**) and the corresponding selection of the largest region. (**f**) Final result, obtained by the application of a region growing process over the selected region in (**e**) to improve the segmented FAZ region and calculate the final FAZ area, shown in μm for more precision.

The FAZ area [13] is automatically extracted from each 3 mm ×3 mm SCP OCTA image, resolution where this region is more clearly identifiable. Firstly, we apply a top-hat morphological operator as preprocessing step, obtaining an enhanced OCTA image. Then, we use an edge detector followed by morphological operators to obtain a binary image with different areas that represents the main existing avascular zones. Given the characteristics of the FAZ region, we coherently select the largest and centered region, posteriorly applying a region growing process to refine the extracted contour. Finally, we calculate the area of this region as the target FAZ area as we see in Equation (1).

(1) $$Area_{FAZ}=p\times \frac{mm^{2}}{H\times W}$$

where *p* represents the number of pixels of the extracted FAZ region, *mm* represents the image size in millimeters, and *H* and *W* indicate the dimensions of the analyzed OCT-A image. To produce a global measurement independent from the resolution, this value represents the area in millimeters. In Figure 4, we can see a representative progressive example of these mentioned steps to obtain the target FAZ segmentation and the corresponding FAZ area measurement.

#### 2.3.2. VD Measurement

In order to extract the VD, we implemented a fully automatic method that precisely segments the vascular regions and measures their constituent density. In particular, this method is based on the analysis of the main structure of the retinal vascularity. Given the high level of noise that is typically present in the OCTA images and the irregularities in its background, we can not use directly the raw OCTA image to measure the VD. In such a case, it is desirable a previous improvement of the analyzed region, reducing the levels of noise and to only preserve the target vascularity. Moreover, it is also remarkable that the OCTA images typically present a high variability in terms of intensities: two OCTA scans taken at the same time and spatial zone of the retina can present variations in terms of intensities. For these reasons, we need a method that contemplates both characteristics: omitting the background noise and being robust to the variability of intensities.

Considering the previous requirements, we first performed a threshold step to segment the higher intensity pixels as vessels by using an adaptive thresholding process [20]. This method provides the robustness that is necessary in this problem given that is based on the intra-class variation minimization. The method organizes the image in two classes where the procedure to obtain the desired threshold between both classes implies an exhaustive search that is based on minimizing the variation intensity in each class and maximizing the variation intensity between both classes. In particular, the equation that is necessary to minimize and consequently obtain this optimal threshold value is the following (Equation (2)):

(2) $$\sigma_{w}^{2}\left(t\right)=\omega_{0}\left(t\right)\sigma_{0}^{2}\left(t\right)+\omega_{1}\left(t\right)\sigma_{1}^{2}\left(t\right)$$

where $\omega_{0}\left(t\right)$ and $\omega_{1}\left(t\right)$ indicate the proportion of pixels in each of the classes generated with the threshold *t*, and $\sigma_{0}^{2}\left(t\right)$, $\sigma_{1}^{2}\left(t\right)$ are the variances of these classes.

**Figure 5:**
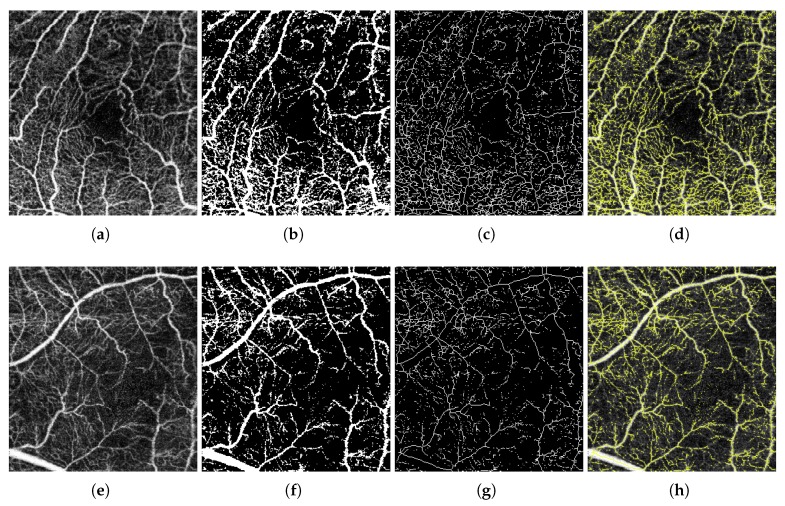
Representative examples of the involved VD extraction steps. (**a**,**e**) Raw OCTA images. (**b**,**f**) Resulting binary OCTA images after applying the adaptive threshold. (**c**,**g**) Skeleton extraction of the binary vascular images. (**d**,**h**) Graphical representation of the zones that are considered for the VD estimation over the original OCTA images.

Once we performed the thresholding process, we obtained a binary OCTA image that represents the zones that contains vascular information. Given that this vascular information still includes representative levels of noise, a further filtering process is necessary to analyze those real pixels of interest. Given the nature of the OCTA images, the main vessels are surrounded by a gradual loss of intensity. This means that the most interesting pixels of the region are the central ones. For this reason, the next step of this method is the skeleton extraction. The skeletonization process is based on a object thinning approach, and it is calculated by the Hit or Miss morphological operator, as we can see in Equation (3), that is iteratively repeated over the binary image until the target skeleton is extracted:

(3) Thinning(I,e)=I∩NOTHit−or−Miss(I,e)

where *I* is the image, *e* is the structural element necessary in the morphological operation and *Hit-or-Miss* is the morphological operator used in the skeleton extraction process. In Figure 5, a few representative examples are presented including the VD extraction steps that we explained before.

Finally, we measure the VD biomarker by the calculus of its proportion over the image, as follows:

(4) $$VD=\frac{p}{height\times width}\times 100$$

where *p* is the count of pixels that are inside of the extracted skeleton, and height and width are the dimensions of the image, measured in pixels.

### 2.4. VA Estimation

To fulfil the final stage of the methodology and perform the automatic VA estimation, we used a Support Vector Machine (SVM) [21] regression model. To do so, we organized the described vascular and avascular extracted biomarkers to, then, be used to train and test the selected model.

#### 2.4.1. Data Preparation

**Figure 6:**
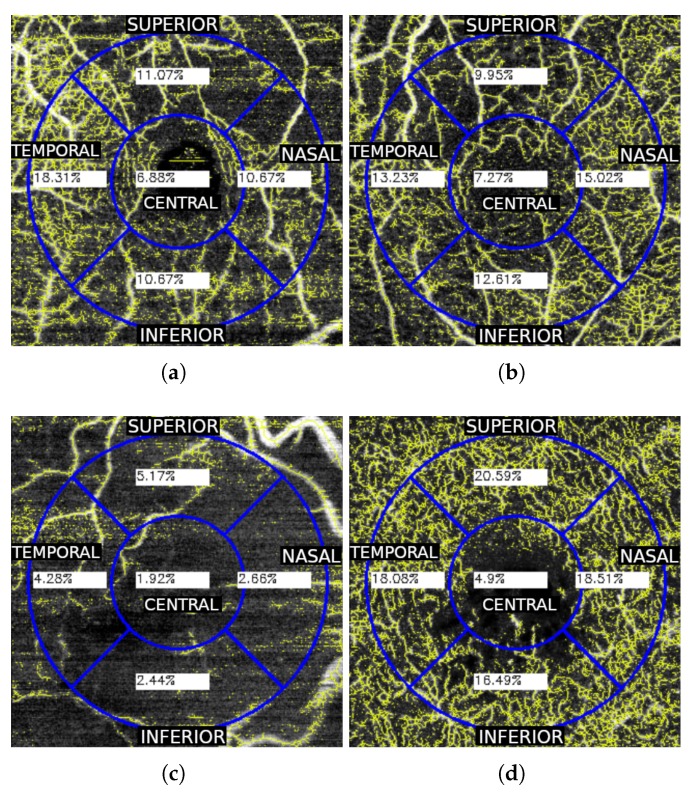
Representative examples of the VD extraction process by the indicated circular quadrant sections in both SCP (**a**–**c**) and DCP (**d**). In these examples we can see different representative degrees of VD loss.

The data that we used to perform the VA estimation are both the automatically measured FAZ area and the VD. In particular, the previously defined VD represents a unique metric that measures the vascular information of an entire OCTA image. However, there are many diseases that present a local impact in particular regions of the eye fundus. For this reason, we decided to expand this metric and calculate the VD in different representative spatial regions of interest of the OCTA images, considering the central one including the fovea center. Also, this decision is reinforced by the mentioned fact that there are different diseases that are represented by the vascular loss in a particular zone. For example, the RVO can be presented more significantly in the superior or inferior regions. Performing the VD as a global metric, the high or low vascularity in a particular zone may loose impact and be diluted in the global metric. On the other hand, if we divide the OCTA image in different representative spatial regions, it is possible to analyze more precisely each one of them. For this reason, we decided to use the traditional circular quadrant grid that is commonly used in the ophthalmic field, analyzing the OCTA images and their corresponding VDs in each of the five quadrants of the grid: central, temporal, nasal, superior and inferior. We show representative examples of the used grid in Figure 6.

In particular, each OCTA image is decomposed into 6 values: FAZ area, central VD, temporal VD, nasal VD, superior VD and inferior VD. As we said, each sample is formed by the information of an unique clinical consultation. This means that each sample takes the data extracted from 4 different OCTA images at 3 mm ×3 mm and 6 mm ×6 mm both in SCP and DCP. It is also remarkable that the VD in temporal and nasal zones are right or left sections depending on which is the pathological eye, therefore we also considered this fact. Given that the FAZ area for SCP and DCP are similar, we only used the extracted FAZ area in superficial mode given its higher precision. In summary, we used 22 features per patient to perform the estimation of the corresponding VA.

However, the experiments were restricted by the limitation of the expert to manually segment some FAZ cases that presented an advanced disease severity. In particular, this means that the expert can not segment the FAZ area given the difficult of the manual segmentation process in these mentioned cases. Given that, in the comparative step, we used both the manual segmentation and our automatic approach, we used the same samples in both methods to perform a reliable and fair comparative. For this reason, in this comparative study, we reduced the used samples from 215 to 127.

#### 2.4.2. SVM Regression Model to Estimate the VA

To perform the VA estimation, we decided to use a SVM regression model. We used the described extracted biomarkers as the input feature vector to extract the predicted VA. In particular, to select the hyper-parameters, we used an automatic exhaustive search to find the best model parameter configuration. This exhaustive search is based on a Cross-Validation (CV) [22] model that minimizes the error function, selecting the parameters that satisfy this minimization. This means that each model is performed with the hyper-parameters that allow the best results with the input data. This is an important characteristic, given that in the experiments we compare the performance of the different methods with the best created model for each input data. For the CV that is performed during the hyper-parameters search, the data was distributed as follows:

- 80% of the dataset was used to perform the CV in the training step with a 5-fold. In this CV, the hyper-parameters were selected, based on the best score over the CV process.
- 20% of the data was utilized to test the model. With the selected hyper-parameters, the model with the best score was selected

## 3. Results and Discussions

Given the complexity of the validation of the VA estimation, we performed different representative complementary experiments for both the biomarkers extraction process and the VA estimation. In particular, our first experiment is based on justifying the use of a regression model with different input features, instead of a direct correlation of a unique feature. In this experiment, we also study the importance of each input feature in the target VA estimation. The second experiment targeted the creation of the comparative model with the semi-automatic extracted VD data of the Topcon capture device and the manually extracted FAZ area of the specialist. Then, we tested the suitability of the automatic extraction of the indicated biomarkers of this proposal, comparing them with the created semi-automatic model as well as the briefly introduced methods of the state of the art.

### 3.1. Experiment 1: Feature Representativity Analysis

#### 3.1.1. Preliminary Individual Feature Analysis

First of all, we performed a previous step of correlation analysis of each identified variable with the target VA, to prove if exits a direct correlation of individual features with our objective. In particular, we used the correlation coefficient [23], that is based on Equation (5), with values between [−1, 1], that represent indirect and direct correlation, respectively.

(5) $$r=\frac{\sigma_{xy}}{\sigma_{x}\times \sigma_{y}}$$

The results of this preliminary study are shown in Table 1. As we can see, the highest correlation value (that is an indirect correlation) is the global VD metric in 6 mm×6 mm OCTA images. This value is justified by the larger analyzed zone of the eye fundus that is presented in these images. However, the coefficient of −0.52 does not represent a significant correlation, it simply indicates that the VD reach high values when the visual loss is low. Moreover, as we said, the OCTA images can include evidences of vascular diseases at inferior, nasal, temporal and superior zones where the global VD does not represent these scenarios correctly. Also, we can see that the central VD in 3 mm×3 mm images presented a correlation of 0.35 with the VA. This correlation is related with the size and shape of the FAZ region and its surrounding vascularity, indications that are coherent with the fact that when the foveal center is affected, the VA is more intensively penalized.

#### 3.1.2. Preliminary Feature Set Analysis

Despite these signs and conclusions, it is clear that there is no significant relation of a single feature with the VA. For this reason, complementary studies were performed to better analyze the significance of the designed feature set.

**Figure 7:**
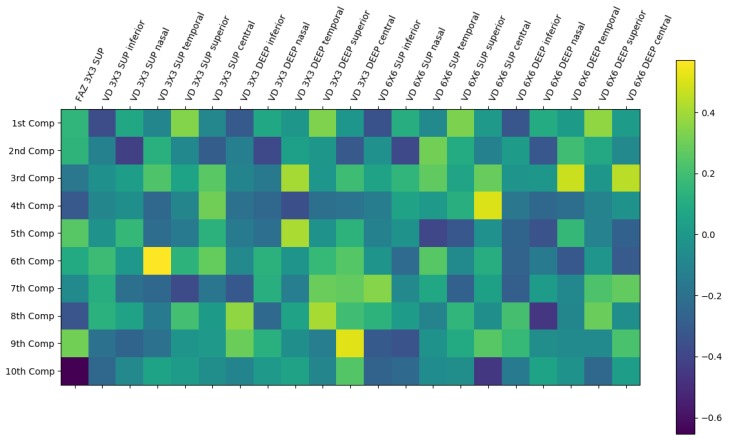
Representation of the significance of each input feature in the extracted Principal Components Analysis (PCA) components. The higher values (yellow cells) represent more significance whereas the smaller values (dark blue cells) represent less significance of the feature in the component.

First, we analyzed the importance of the features using a Principal Components Analysis (PCA) [24]. We obtained, after applying this method, that 10 constructed components explain the 89.5% of the variance of the model, as we can see in Table 2, which presents the explained variance per component. This implies that we would need a large number of components, and this means that the faced problematic is not easily resolved directly with a small set of features. In Figure 7, we can see the graphical representation of the significance of each input feature in the created components. In this representation, the higher values (green and yellow cells) represent more significance of the features in the component. In particular, we can see that there is no feature that could be removed from the set, given that they present a high significance in, almost, one component. This reinforces the complementary utility and significance of the extracted biomarkers in the analysis of the VA.

We also performed a feature study using the Correlation Feature Selection (CFS) [25]. This method is based on measuring the correlation of the features with the regression target by three different measurements of relation instead of the traditional correlation: Minimum Description Length [26], Mutual Information [27] and relief [28]. The method, after applying these algorithms, provides a score for each feature, as well as a *p*-value that represents the reliability of that score. In Table 3, we can see the results after applying the CFS method to our designed feature set. Hence, the highest obtained values remain in the 6 mm ×6 mm OCTA images, explained by the fact that they represent a larger zone than 3 mm ×3 mm and the information that they provide is also more relevant. In particular, the highest score is reached in the VD at the center of the 6 mm ×6 mm DCP OCTA images that, as we said, is determined by the FAZ regions and their surrounding vessels, as well as their irregularity. Once again, the statistics corroborate the relevance of the foveal center region in relation with the VA.

With this preliminary analysis, again, we can conclude that the problem is not easy to solve directly as an accurate combination of representative extracted features is required to estimate the VA. Moreover, the central VD of the DCP in 6 mm ×6 mm provides better information about the VDs from the other regions. This score is also justified by the high variability of the central zone in this type of image, representing both the FAZ and its surrounding vessels. Hence, this metric provides a higher score in the features with more variability, given that the higher the variability is, the more information is reached. This also corroborates the clinical knowledge that the more centered in the fovea a pathological condition is, the higher the vision loss is. Another interesting fact that better representativity of the VD biomarker in DCP instead of SCP OCTA images indicates that damages and alterations in the deep vascularity produces a more significative impact in the VA of the patients.

### 3.2. Experiment 2: Comparative with the State of the Art and the Created Model Using the Capture Device Information

#### 3.2.1. Validation Metrics

Once the dataset was prepared, we adjusted the estimation model, and selected the hyper-parameters. To do so, we used, as we said, an exhaustive search of the best parameters for the input data. Hence, the obtained model with the selected hyper-parameters is employed to perform the VA estimation, measuring the error between the manually calculated VA and the computational estimated VA. This measurement was performed using the Mean Absolute Error (MAE) and the Root Mean Squared Error (RMSE). The MAE and RMSE were calculated following Equations (6) and (7).

(6) $$MAE\left(y,\hat{y}\right)=\frac{1}{n}\sum_{i=0}^{n-1}\left|y_{i}-{\hat{y}}_{i}\right|$$

(7) $$RMSE\left(y,\hat{y}\right)=\sqrt{\frac{1}{n}\sum_{i=0}^{n-1}{\left(y_{i}-{\hat{y}}_{i}\right)}^{2}}$$

where *n* is the number of samples, *y* is the manually calculated value of the VA and $\hat{y}$ is the estimated value of the VA by the proposed method.

We decided to perform both metrics given that we can extract complementary information from there. In particular, MAE provides information about the mean error between the manually extracted and the predicted values, while RMSE penalizes more significantly the larger differences by the quadratic calculation. Moreover, the VA is a metric that is measured between values of 0 and 1 as a logarithm in base 2 interpretation, but we convert this value to a logarithm in base 10 scale to allow the use of equidistant values. However, this conversion does not change the range value of the variable but facilitates the estimation process.

#### 3.2.2. Capture Device Biomarker Estimation

Given the novelty of the method, there exist very few alternative proposals in the state of the art [12] that measure the FAZ and the VD to perform a robust comparative with the automatic measurements of this work. In fact, this calculation and performance comparative needs to be accurate, given that the calculation of the presented biomarkers is critical for the posterior estimation of the VA. For this reason, we calculated biomarkers of reference for the comparative using the semi-automatically extracted data from the Topcon capture device. In particular, this device provides the option of manually segmenting the FAZ, calculating posteriorly the FAZ area. Moreover, the device allows the initialization of the VD calculation, after providing the manual initialization of the FAZ center. This process is, obviously, not automatic and needs the subjective intervention of the specialists, but serves as reference for the comparative study. A further limitation is that the capture device only handles the SCP OCTA images, given the difficulty of the DCP cases. Also, the FAZ area can only be measured in 3 mm×3 mm SCP and in several cases the expert could not estimate the FAZ manual segmentation. Therefore, the number of samples was restricted from 215 to 127, as we said.

Firstly, we prepared the data as we explained before: each sample contains the extracted information from an unique medical consultation. In this case, we used the extracted biomarkers from both 3 mm×3 mm and 6 mm×6 mm SCP OCTA images for the comparative study. Comparing the obtained results with the reference performance, we obtained values of MAE and RMSE of 0.2419 and 0.3034, respectively, with an increment of the RMSE over the MAE of 20.32%, as detailed in Table 4. This implies that there are some VA estimations that are more distant of the device performance than the MAE. This could be justified by the manual component of the semi-automatic biomarkers. However, instead of the extracted values (by the Triton Topcon capture device in this case), it is relevant that the VA is also calculated by a manual mode, that can include wrong measurements in some cases, introducing noise to the model, which increases the obtained errors.

#### 3.2.3. VA Estimation Using the FAZ Area and the VD Extracted Biomarkers

Given that the measurements that form the created semi-automatic model are only extracted from SCP OCTA images, we performed two estimation approaches:

- A first VA approach with the same corresponding features of the semi-automatic created model to perform a reliable and fair comparative.
- Then, we performed a second VA approach using the data extracted by the automatic proposed method using both SCP and DCP OCTA images in order to employ all the possible automatically extracted information in the VA estimation process.

In both cases, we trained the model with the best configuration of their hyper-parameters, as we indicated before.

In Table 4, we can see the obtained results, including values of 0.2338 and 0.2848 for MAE and RMSE, respectively, in the trained model with the SCP OCTA extracted data, demonstrating that our measured biomarkers are more precise than the semi-automatically extracted ones using the capture device. Consequently, these results also confirmed that our approach is more reliable to perform the VA estimation. In particular, observing the column increment in Table 4, we can see that the difference between the RMSE and the MAE is lower in our approach than in the constructed baseline model.

This means that our model has fewer incorrect estimations, which can be explained by the fact that our method excludes many irrelevant areas of the image that may alter the measuring process given the existing noisy levels, contrary to the performance of the capture device.

Additionally, the results using the extracted data from the SCP and DCP images were 0.1713 and 0.2354 for MAE and RMSE, respectively, giving a better VA estimation than the one obtained using the constructed ground-truth. This mainly demonstrates that the information that is also contained in DCP OCTA images is relevant to estimate the VA metric. However, in the increment column we can see that there exists a higher difference in the MAE and RMSE than in the previous experiment only using the SCP OCTA data. This means that the DCP OCTA images include a higher quantity of noise in their capture process, which significantly hardens the automatic vascularity identification. In summary, the DCP OCTA data provides better conditions for the VA estimation but also introduces some noise to the process.

#### 3.2.4. Overall Comparison

Finally, we performed an extended experiment that involved all the mentioned methods: the briefly introduced methods of the state of the art, the created semi-automatic model and our automatic approach. In the experiment, we compared the applied MAE and RMSE over each created model that represents each of the compared methods and the manual measurement of the VA. In particular, the briefly introduced methods in the state of the art [12] are explained as follows:

- Original: computing the VD over the raw OCTA image.
- Binary: applying a fixed threshold to the input OCTA image before the VD extraction.
- Weighthed: computing the VD by increasing the importance of the pixels below a threshold.
- Skel: extracting the skeleton over the binary image and computed the VD over its skeleton.

For each of these four methods, we trained two differentiated models: a model using the extracted data in SCP, to compare with the created baseline of the capture device, and another more complex model based on the VA estimation using both SCP and DCP fully automated extracted data. The created semi-automatic model extracted with the capture device, as we said, is limited by the fact that the capture device only manages the SCP OCTA images. For this reason, we created a model that only uses the information that was semi-automatically extracted by the SCP OCTA images. It is remarkable, as we said, that this approach is constructed with a fully manually segmented FAZ, and a manual initialization of the FAZ center to semi-automatically determine the VD in each circular quadrant. Finally, our approach automatically extracts both the FAZ area and the VD, and we created a model that estimated the VA with all of these previously extracted biomarkers.

In Table 4, we can see the results of MAE and RMSE after training each model and performing the test validation for all the six analyzed methods. As we can see, the four previously introduced methods are too imprecise to estimate an accurate VA. This is explained by the fact that these methods did not introduce the precondition of selecting an area with a lower noise level, and the measurement was performed with pixels that do not belong to vessels. In fact, the method with the lowest MAE is the *Skel* because that it is the method that uses fewer pixels from the image to perform the VD, and consequently, the metric contains lower noise levels. It is also remarkable that in all these four cases, the DCP deteriorates the performance of the methods. This supports the fact that these methods typically include a higher number of noisy zones in the OCTA images, consequently deteriorating the VA estimation. In fact, from the previous four approaches, only the *skel* method improves the performance by adding the analysis of DCP OCTA images, given that, as we said, it is the method that contains lower noisy levels. Additionally, the availability of SCP and DCP extracted data certainly improves the input information and the corresponding VA estimation of the model.

The RMSE increment over the MAE in these four experiments is lower than in our approach and the created semi-automatic baseline. This implies that in these four methods the MAE is high. As we said, the created baseline is obtained by the FAZ manual extraction and the semi-automatic VD extraction. Both metrics were extracted by an expert clinician and only in the SCP data to, then, perform the VA estimation process. As we can see in the table (Table 4), the obtained MAE and RMSE were slightly higher than the obtained ones with our approach. Moreover, the RMSE increment over the MAE implies a higher variability in the error estimation than in our approach. Finally, our proposed approach automatically extracts both the FAZ area and the VD to perform the VA estimation process. We also present in Table 4 the results using both the SCP and DCP OCTA images improving, as shown, the obtained performance of the previously created baseline.

As we can see, our method provides an accurate approach with a lower level of noise that is justified by the improvement of the results after including the DCP data, which is characterized by containing a higher level of noise. It is also remarkable that the inclusion of the DCP data is highly relevant in this problematic, given that it provides an amount of information that allows us to greatly improve the results of both MAE and RMSE. This means that the DCP OCTA images include significant noisy levels but the damage and alterations in the deep vascularity are intimately linked to pathologies and the consequent loss of the VA.

## 4. Conclusions

Many systemic and eye diseases affect the retinal micro-circulation and the characteristics of its vascularity. Hence, RD is one of the main diseases that may cause ocular micro-circulation problems. It is characteristic of regional vascular deterioration in the advanced stages of the pathology, as well as the consequent gradual vision loss. Also, ocular diseases such as RVO, for reference, may deteriorate the eye micro-circulation, with the corresponding visual penalization. In that context, early pathological identification is crucial but challenging. The disease is commonly detected in advanced stages when the clear symptoms are experienced. For these reasons, it is necessary to detect these vascular diseases in their initial stages by identifying slight changes in the retinal vascular circulation in order to avoid the drastic consequences of the advanced pathological stages and revert the condition.

The principal pathological changes in the vascular structure are mainly represented by the loss of vascular zones in the retina. In this context, the recent appearance of the OCTA image modality and its significant increase in popularity is motivated by its characteristic detailed visualization of the vascular structure of the eye fundus, representing an accurate source of information for the analysis of the retinal vascular diseases. However, the manual analysis of this vascular structure is tedious and requires a time-consuming process.

Commonly, the visual loss is measured by the VA of the patient, which is typically extracted by different manual tests with the patient and the expert clinician. The VA extraction is a slow process and it is conditioned by the non deterministic reply of the patient and the assessment of the clinician. The fast, deterministic and objectivity factors that generally provide an automatic approach are desirable in this problem.

In the present work, we propose a novel fully automatic methodology to estimate the VA of the patient, which is supported by the extraction of representative vascular and avascular biomarkers captured from the OCTA images. In particular, we measure representative biomarkers as the FAZ area and the VD. We use them as genuine biomarkers to estimate the VA of the patient, dividing the analysis of the VD into 5 circular quadrants to improve the precision of the information provided by the input features of the VA estimation model. Given that the VD extraction process removes all the possible regions that are most certainly not vessels, our proposal is robust to the presence of noise or motion artifacts. Also, the fact of using 5 different circular quadrants to create the feature vector that is used to estimate the VA provides a positive tolerance to particular local imperfections in the capture process. In the case that any significant noise is included in any region of the circular quadrant, this region only represents 1 of the 21 total used features to estimate the VA, given that we are also using four complementary OCTA images to perform this estimation. In summary, this means that the method is highly robust and tolerant to the presence of noise and artifacts in the OCTA images, which is still a common situation in this novel image modality.

Then, to perform the validation process, we compared our approach with the state of the art and the created capture device model, based on the existing Topcon semi-automatic tools. With these comparisons, we demonstrated that our VD extraction provides robust results to perform the VA estimation that can be accurately combined with the analysis of the FAZ area. The target VA is estimated with an error of 0.1713, providing an accurate system to evaluate the visual loss of the patient. This way, the proposed method offers automatically the estimation of the VA of the patient extracting representative computational biomarkers related with the FAZ region and the VD.

As future work, we propose the improvement of this VA estimation by adding new complementary biomarkers from the OCTA images. Additionally, it would be interesting to extend the analysis of the VA to patients with other relevant diseases and measure the relation of the vision loss and the vascular and avascular characteristics of the eye fundus. Indeed, it would also be interesting to use this estimation complementarily to other extracted biomarkers to obtain a model that aids in the clinical diagnostic process in the clinical field using a novel image modality as is the case of OCTA.

## Figures and Tables

**Table 1:** Coefficient of correlation of each variable with the VA.

Coef. Correl.	3 mm ×3 mm SCP	6 mm ×6 mm SCP	3 mm ×3 mm DCP	6 mm ×6 mm DCP
FAZ area	0.1695	-	0.0206	-
Global VD	0.1230	−0.2432	−0.1265	−0.5200
Inferior VD	−0.1260	−0.2947	−0.1938	−0.2712
Nasal VD	0.0974	−0.1119	−0.1305	−0.2901
Temporal VD	0.0160	−0.1458	−0.0597	−0.3128
Superior VD	0.1283	−0.0712	−0.0712	−0.1863
Central VD	0.3511	−0.0138	0.3520	−0.2468

**Table 2:** Explained variance of each extracted PCA component.

Component	1	3	2	4	5	6	7	8	9	10
**Explained Variance**	0.2476	0.1785	0.1158	0.0894	0.0659	0.0582	0.0498	0.0379	0.0346	0.0272

**Table 3:** Correlation Feature Selection (CFS) application over the used dataset, obtaining as a result the score of each feature in the regression problem, as well as their reliability *p*-Value.

CFS	3 mm ×3 mm SCP		3 mm ×3 mm DCP		6 mm ×6 mm SCP		6 mm ×6 mm DCP	
	Scores	*p*-Value	Scores	*p*-Value	Scores	*p*-Value	Scores	*p*-Value
FAZ area	1.5890	0.209	-	-	-	-	-	-
Inferior VD	0.0340	0.854	1.5091	0.221	5.0846	0.026	6.1905	0.014
Nasal VD	1.2398	0.267	1.4066	0.237	7.5289	0.006	0.4078	0.524
Temporal VD	0.0736	0.786	0.6975	0.405	6.4929	0.012	6.3829	0.012
Superior VD	0.2560	0.613	0.2879	0.592	6.8191	0.010	3.7112	0.056
Center VD	5.2321	0.023	2.7808	0.098	0.6450	0.423	14.5189	2.17 $\times \;10^{-4}$

**Table 4:** Comparison with the state of the art and the created baseline.

Approach	SCP			SCP & DCP		
	MAE	RMSE	Increment	MAE	RMSE	Increment
Original	0.2791	0.3121	10.57% ↑	0.3188	0.3681	13.39% ↑
Binary	0.2690	0.3074	12.49% ↑	0.2859	0.3148	9.18% ↑
Weighted	0.2730	0.3076	11.24% ↑	0.2989	0.3458	13.56% ↑
Skel	0.2783	0.3145	11.51% ↑	0.2338	0.2703	13.50% ↑
Created baseline	0.2419	0.3036	20.32% ↑	-	-	-
Our approach	0.2338	0.2848	17.90% ↑	0.1713	0.2354	27.23% ↑

## Abbreviations

The following abbreviations are used in this manuscript:

OCT	Optical Coherence Tomography
OCTA	Optical Coherence Tomography by Angiography
VA	Visual Acuity
SVM	Support Vector Machine
SCP	Superficial Capillary Plexus
DCP	Deep Capillary Plexus
RVO	Retinal Vein Occlusion
DR	Diabetic Retinopathy
FAZ	Foveal Avascular Zone
VD	Vascular Density
CFS	Correlation Feature Selection
MAE	Mean Absolute Error
RMSE	Root Mean Squared Error
CAD	Computer Aided Diagnostic
